# Characterization of a new physical phantom for testing rigid and deformable image registration

**DOI:** 10.1002/acm2.12514

**Published:** 2018-12-23

**Authors:** Richard Y. Wu, Amy Y. Liu, Paul Wisdom, Xiaorong Ronald Zhu, Steven J. Frank, Clifton D. Fuller, Gary Brandon Gunn, Matthew B. Palmer, Cody A. Wages, Michael T. Gillin, Jinzhong Yang

**Affiliations:** ^1^ Department of Radiation Physics The University of Texas MD Anderson Cancer Center Houston TX USA; ^2^ Department of Radiation Oncology The University of Texas MD Anderson Cancer Center Houston TX USA; ^3^ Dosimetry Service The University of Texas MD Anderson Cancer Center Houston TX USA

**Keywords:** deformable phantom, multimodality image registration, rigid and deformable image registration

## Abstract

The purpose of this study was to describe a new user‐friendly, low‐cost phantom that was developed to test the accuracy of rigid and deformable image registration (DIR) systems and to demonstrate the functional efficacy of the new phantom. The phantom was constructed out of acrylic and includes a variety of inserts that simulate different tissue shapes and properties. It can simulate deformations and location changes in patient anatomy by changing the rotations of both the phantom and the inserts. CT scans of this phantom were obtained and used to test the rigid and deformable registration accuracy of the Velocity software. Eight rotation and translation scenarios were used to test the rigid registration accuracy, and 11 deformation scenarios were used to test the DIR accuracy. The mean rotation accuracies in the X‐Y (axial) and X‐Z (coronal) planes were 0.50° and 0.13°, respectively. The mean translation accuracy was 1 mm in both the X and Y direction and was tested in soft tissue and bone. The DIR accuracies for soft tissue and bone were 0.93 (mean Dice similarity coefficient), 8.3 and 4.5 mm (mean Hausdouff distance), 0.95 and 0.79 mm (mean distance), and 1.13 and 1.12 (mean volume ratio) for soft tissue content (DTE oil) and bone, respectively. The new phantom has a simple design and can be constructed at a low cost. This phantom will allow DIR systems to be effectively and efficiently verified to ensure system performance.

## 1 INTRODUCTION

In the past decade, many commercial software programs have been developed to perform deformable image registration (DIR) in clinical use. The technological developments have called for meticulous quality assurance procedures before these systems can be used in the clinic. At present, most efforts to test DIR accuracy have included landmarks or contours to estimate the error in the displacement of the vector field of the DIR.^1^, ^2^, ^3^, ^4^ However, these evaluations are limited by the number of objects being tracked.^5^ A library of virtual phantoms was introduced by Pukala et al.; these were intended to resemble real cases for the head and neck DIR tests^6^ but were only applicable to cases involving the same treatment characteristic (e.g., site and imaging modality). Stanley et al. developed a patient‐specific computational model phantom^7^ for the prostate gland and lungs that was mainly based on the finite element modeling framework. Used as ground truth to test a DIR system, it is possible the deformation may be limited and the test may be biased toward the finite element‐based registration algorithms. Yu et al. used an approach to identify the anatomical changes that occur during radiation therapy and created active shape models to generate artificial CT images with known deformation.^8^ This approach is limited by the registration errors of the DIR algorithm that was used to create the training displacement vector fields, including the interpatient and intrapatient registration errors.

Normally, a physical phantom is required to accomplish a complete end‐to‐end evaluation of the accuracy of image acquisition and the DIR process. The phantom defines the dimensions and characteristics that are used to test the DIR metrics under a variety of conditions. Later on, a motor‐controlled deformable physical phantom was designed to test lung tissue deformation accuracy.^6^ However, the phantom was too large to be practical in a clinical environment because the scale of deformation could not be accurately quantified. Wongnum et al. developed a porcine bladder phantom that simulated bladder changes that could occur during the course of radiation treatment.^9^ However, this phantom suffered from storage problems after each use and lacked quantitative information. Singhrao et al. developed a deformable head and neck phantom using thermoplastic materials that mimicked head and neck patient anatomy.^10^ This phantom used optical markers to measure deformation from the coordinated information extracted from an optical camera via in‐house software. To generate deformation, pressure was applied to the back of the phantom. The authors did not intend for their model to be used as an end‐user phantom since the use of the phantom and characterization of the deformation require a considerable amount of time and expertise. Kirby et al. developed a pelvic phantom that used rubber, mineral‐filled plastic, and nylon bolts to simulate a real patient.^11^ However, the phantom required special knowledge and materials that are not available to all DIR users. Other anthropomorphic phantoms have been developed for quality assurance,^12^, ^13^ but they have not had the ability to simulate a variety of tissue deformations and location changes. A validation method based on physician‐drawn structure contours or physician‐picked anatomical landmarks has also been widely used.^14^ However, this approach is time‐consuming and inevitably suffers from interobserver and intraobserver variability. Recently, a new virtual phantom was published in the American Association of Physics in Medicine (AAPM) task group 132 report, which can be downloaded online, to test DIR accuracy.^15^ This virtual phantom has several limitations. It uses image offset instead of physical phantom movement to test the rotation, translation, orientation accuracy. Therefore, the end‐to‐end test of accurate data representation, image transfer, and integrity verification between image acquisition devices and image registration systems cannot be performed. The phantom also uses fixed insets and shapes of which a contour deformation cannot be simulated and the test of DIR accuracy cannot be simultaneously performed under rotation, translation, and deformation conditions. In addition, the DIR test used an anthropomorphic pelvis phantom, which has limited image contrast. The high (lung) and low (brain) contrast subjects were not included and cannot be validated under such clinical conditions. Although there are numerous methods to independently validate DIR systems, all of them demand a great deal of resources and time.

The purpose of this study was to design and develop a user‐friendly, low‐cost physical phantom that would be capable of testing rigid and DIR accuracy in a streamlined and seamless fashion (provisional patent application, Attorney Docket No.: UTSC.P1357US.P1). The phantom was constructed using a variety of inserts simulating different shapes and properties of human tissue. These inserts can be arranged to simulate rigid or deformable changes in the patient anatomy as compared with its reference position. This phantom has labeled dimensions, which facilitates quantitative measurements for accuracy tests of both rigid and deformable registration. It can be imaged with CT, MRI, and PET/SPECT scanners to test DIR accuracy of multiple imaging modalities. Users can also use different materials in the inserts to test the DIR accuracy in a wide variety of clinical situations using both high‐ and low‐contrast media.

## 2 MATERIALS AND METHODS

### 2.A Phantom design

**Figure 1:**
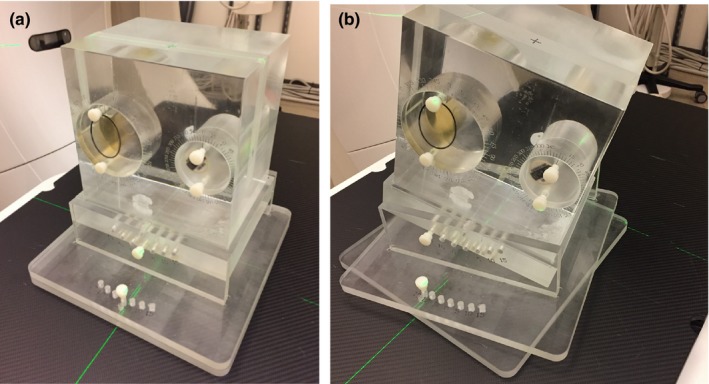
The Wuphantom is made of acrylic with two insert cavities and a base that allows tilting and rotation. (a) Reference position. (b) The base has 15° of tilting and rotation.

**Figure 2:**
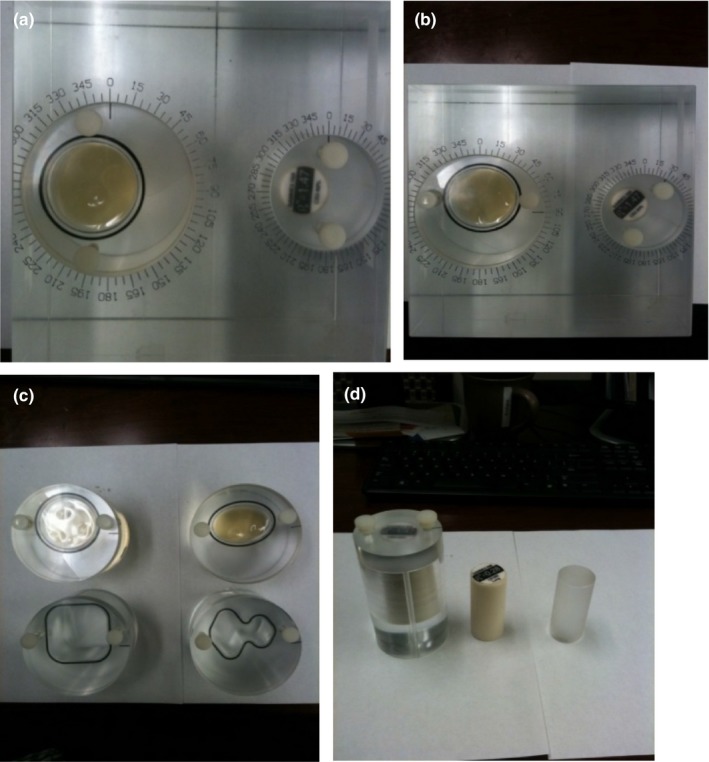
(a) Engraved marks indicate the degree of the rotation of inserts. Two inserts can be filled with different materials to simulate a change in tissue. The large insert on the left is filled with DTE oil (ExxonMobil, USA). The small insert on the right is a bone plug from an RMI phantom. (b) After 90° of rotation for both large and small inserts, the center location of both inserts changed compared with the reference location (without rotation). (c) Large cavity inserts were created in three different shapes: circle, oval, and tree. A rectangular insert was created for future use. (d) RMI inserts with known electron densities that can facilitate the test of the location change of different types of tissue. Soap and oatmeal were used in the insert when performing the DIR test simulating different tissue characteristics.

The design of the phantom (called Wuphantom, named after the designer) is shown in Fig. 1(a). The main body of the phantom is fabricated from acrylic plastic with a density of 1.02 g/ml, which is slightly heavier than water. A phantom holder was constructed to allow tilting and rotation [Fig. 1(b)]. There are two round insert slots [Fig. 2(a)] with engraved marks to indicate the rotation (in degrees) of the inserts. The two inserts have internal cavities that can be filled with different materials of interest. A cap with a rubber seal is provided to enclose the inserts to prevent leakage. The insert internal cavities are located off the central axis so that when the inserts are rotated, the position of the center will simulate changes in contour location due to body deformation [Fig. 2(b)]. The Wuphantom has overall dimensions (cm) of [20 L × 20 W × 15 H (depth)].

Three large cavity inserts were made in different shapes: circle, oval, and tree, simulating deformed contours from the original circle [Fig. 2(c)]. The oval shape represents commonly deformed contours, and the tree shapes can simulate irregularly deformed contours. The phantom can be tilted and rotated to test rigid image registration. Each of the inserts can also be rotated to simulate contour changes in both shape and location compared to the reference circle, which is usually used as the reference. Each insert cavity can be independently filled with solid or liquid materials with different densities, simulating different types of tissue in patients. A smaller cavity was constructed on the right side of the phantom to accommodate commercially available RMI inserts (Gammex, Inc.) with known densities [Fig. 2(a) and 2(d)] and test the location changes of different types of tissue with known electron densities. We performed quality assurance on the phantom construction. The phantom rotation and tiling angle were within 0.12° of accuracy.

### 2.B Phantom image acquisition

Images of the Wuphantom were acquired using a Siemens Definition Edge computed tomography (CT) scanner with a voxel resolution of 0.98 × 0.98 × 2 mm. The scanning was performed using the established head and neck CT protocol (35 cm FOV, 120 kvp, 2.0 mm slice thickness, and 300 mA). The CT images were then transferred to a Velocity Workstation (Varian, Inc.). To obtain the reference image, the phantom was placed on the base with the large circle insert filled with Mobile DTE oil and the small circle insert filled with a bone plug (CB2 30%) from an RMI phantom (Gammex, Inc.). Mobile DTE oil has a density of 0.95 g/ml, representing soft tissue. The bone insert has a density of 1.33 g/ml. All of the alignment marks (insert rotation, phantom tilting, and rotation) were set at 0° during the acquisition of the reference image set. Finally, contours were delineated for large and small inserts using a predefined threshold CT# range.

### 2.C Image registration tests

#### 2.C.1 Rigid image registration test for rotation and translation

**Figure 3:**
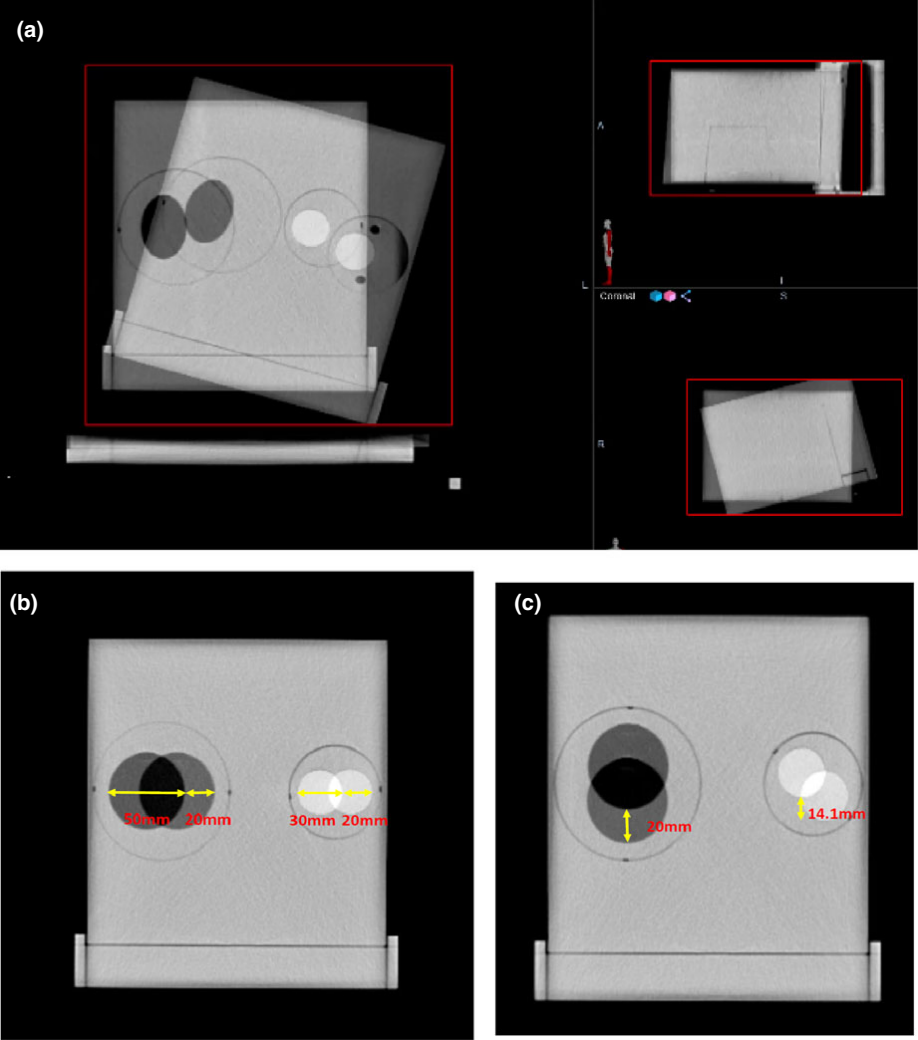
(a) The phantom had 15° of tilting and 15° of rotation. (b) The large and small circular inserts were rotated 180° to create a 2‐cm location change in the X direction compared to the reference image (overlaid). (c) The large and small circular inserts were rotated 270° and 225°, respectively, to create a 2‐cm location change in the Y direction for the large circle and 1.41 cm for the small circle compared to the reference image (90° and 45° overlaid).

After the reference image scans were taken, a new phantom image set (also called secondary images) was acquired after tilting and rotating the base by 5° and 15° counterclockwise, respectively [Fig. 3(a)], to test the system's rigid registration under the predetermined degrees of angle of rotation. For translation accuracy tests, another set of phantom secondary images was acquired by rotating both the large circle and the small circle inserts to create a location change of the insert in the X and Y directions for comparison with the original reference images. For these tests, the large insert was filled with DTE oil and the small insert was filled with a bone plug for simulating location changes for soft tissue and bone.

For the X direction movement test, the large insert (DTE oil) and small insert (bone) were rotated from 0° to 180° relative to the reference position. This produced a distance displacement of 20 mm in the X direction for both density inserts used [Fig. 3(b)]. For testing movement in the Y direction, the large insert was rotated from 270° to 90°, and the small insert was rotated from 225° to 45°, simulating displacements of 20 and 14.1 mm, respectively, in the Y direction. The images were first roughly registered using manual alignment by shifting and rotating the secondary image. A region of interest that encompassed the whole phantom was drawn. The Velocity rigid registration process was used to align the two image sets.

#### 2.C.2 Deformed image registration test

**Figure 4:**
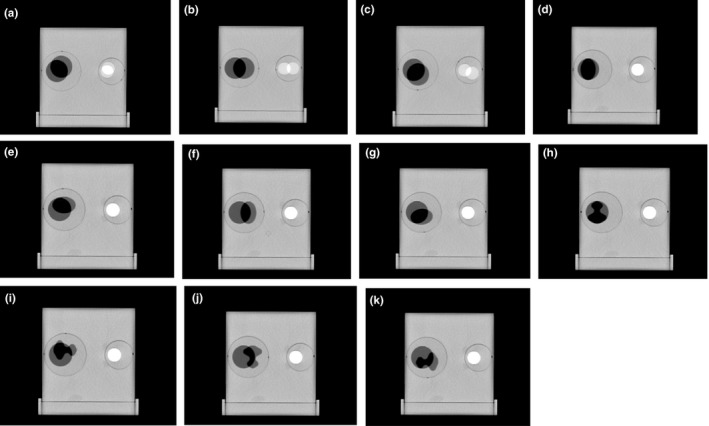
The contour deformation and location change were simulated by changing and rotating the inserts. (a) Circle rotation = 90°, bone rotation = 90°. (b) Circle rotation = 180°, bone rotation = 180°. (c) Circle rotation = 270°, bone rotation = 225°. (d) Oval rotation = 0°, bone rotation = 0°. (e) Oval rotation = 90°, bone rotation = 0°. (f) Oval rotation = 180°, bone rotation = 0°. (g) Oval rotation = 270°, bone rotation = 0°. (h) Tree rotation = 0°, bone rotation = 0°. (i) Tree rotation = 90°, bone rotation = 0°. (j) Tree rotation = 180°, bone rotation = 0°. (k) Tree rotation = 270°, bone rotation = 0°.

The secondary images for the DIR accuracy tests were acquired by replacing and rotating the circular and oval‐ and tree‐shaped inserts. This was done to simulate tissue deformation from a circular shape to another circular shape or an irregular shape (oval or tree). Rotating the inserts to a different degree simulates the location changes for the contours of interest. Shown in Fig. 4 are 11 combined contour deformation scenarios that simulate both contour deformation and location changes. The small circle (filled with the bone plug) had both rotation and nonrotation conditions, simulating the clinical condition in which only soft tissue had deformation and there was no bone deformation. We used the rigid and deformable multipass tool in the Velocity software program to perform the DIR process for all the selected secondary image sets. All of the corresponding contours were propagated into the secondary image sets.

### 2.D Accuracy of DIR

The accuracy of the DIR of a contour can be characterized by three major factors: the conformity index (also called the Dice coefficient index), the maximum surface distance (also called the Hausdouff distance), and the volume ratio. The conformity index is defined as the ratio of twice the overlap of two structures over the sum of their volumes. This method is widely used in DIR comparisons.^16^ The conformity index ranges from 0 to 1, denoting the degree of a perfect match between the two structures:

(1) $$D\left(A,B\right)=2\left|A\;and\;B\right|/\left(|A|+|B|\right)$$

where A and B are the two sets. More simply, this formula represents the size of the union of two sets divided by the average size of the two sets. A value of 0 indicates no overlap, and a value of 1 refers to perfect agreement. Higher numbers indicate better agreement. The maximum surface distance is the greatest of all the distances from a point in the original contour to the closest point of the deformed contour:

(2) $$h\left(A,B\right)=\max_{a\in A}\left\{\min_{b\in B}\left\{d\left(a,b\right)\right\}\right\}$$

where a and b are points belonging to sets A and B, respectively. The volume ratio is defined as the ratio of the deformed volume to the reference volume:

Volumeratio=deformedvolume(cc)/referencevolume(cc)

## 3 RESULTS

Table 1 shows the results of rigid registration tests for eight combined scenarios of the phantom and base rotations. The mean absolute difference between the measured and indicated angles were 0.5° and 0.13°, respectively, for the tilting and rotation. Test results for the phantom translation cases are displayed in Table 2. The mean difference in the measured distance between the movement of the large circle (DTE oil) and small circle (bone) was 1 mm in the X and Y directions. For the DIR test results shown in Table 3, the mean conformity index value was 0.93 for soft tissue contours (circular and oval‐ and tree‐shaped inserts filled with DTE oil) and bone contours. Similarly, the maximum surface distances were 8.3 mm for soft tissue contour and 4.56 mm for bone. The mean surface distances were 0.95 and 0.79 mm for soft tissue and bone, respectively. The mean volume ratios between the deformed volume and the reference volume were 1.13 for soft tissue and 1.12 for bone. The volume ratio is slightly higher at 270° than 90° for circle‐ and olive‐shaped inserts and slightly lower for tree‐shaped inserts (1.11 vs 1.08). We believe this may be due to the magnitude change of location after the deformation of contours. It should be noted that all of the accuracy test results were well within the range recommended by medical physics community and the AAPM TG 132 report.^15^

**Table 1:** Phantom rotation accuracy test results of rigid registration

Rotation test						
	Indicated tilting angle (degree)	Measured tilting angle (degree)	Absolute difference (degree)	Indicated rotation angle (degree)	Measured rotation angle (degree)	Absolute difference (degree)
Reference T0B0	0	0	0	0	0	0
T5B0	5	5.6	0.6	0	0.0	0.0
T15B0	15	15.2	0.2	0	0.0	0.0
T0B5	0	0.6	0.6	5	5.0	0.0
T0B15	0	0.1	0.07	15	15.1	0.1
T5B5	5	5.5	0.5	5	5.1	0.1
T5B15	5	5.7	0.7	15	14.9	0.1
T15B5	15	15.4	0.4	5	4.8	0.2
T15B15	15	15.9	0.9	15	14.5	0.5
Mean			0.50			0.13

T, phantom tilted degree; B, phantom base rotation degree.

**Table 2:** Phantom translation test results of rigid registration

Translation test					
	Large circle moved distance from insert rotation (mm)	Measured large circle moved distance (mm)	Small circle moved distance from insert rotation (mm)	Measured small circle moved distance (mm)	Mean difference (mm)
X direction	20	19.6	20	18.4	1.0
Y direction	20	20.8	14.1	15.3	1.0

**Table 3:** Deformable image registration (DIR) accuracy testing results

Rigid + deformable multipass												
	Conformality (Dice)		Surface distance metrics (mm)				Volume test					
			Max (HD)		Mean		Reference volume (cc)	Deformed volume (cc)	Volume ratio (deformed/reference)	Reference volume (cc)	Deformed volume (cc)	Volume ratio (deformed/reference)
Reference circle 0 Bone 0	Circle/olive/tree	Bone	Circle/olive/tree	Bone	Circle/olive/tree	Bone	Circle	Circle/olive/tree		Bone	Bone	
Circle 90 Bone 45	0.95	0.93	5.80	4.80	0.64	0.87	91.90	100.60	1.09	44.40	49.60	1.12
Circle 180 Bone 180	0.92	0.85	5.80	7.80	1.29	1.71	91.90	108.40	1.18	44.40	59.30	1.34
Circle 270 Bone 225	0.95	0.90	6.90	11.70	0.59	1.20	91.90	100.30	1.09	44.40	52.50	1.18
Olive 0 Bone 0	0.96	0.97	8.70	2.93	0.47	0.45	91.90	98.20	1.07	44.40	47.30	1.07
Olive 90 Bone 0	0.95	0.94	7.80	4.00	0.54	0.74	91.90	100.30	1.09	44.40	49.20	1.11
Olive 180 Bone 0	0.93	0.96	6.26	2.18	1.08	0.52	91.90	100.60	1.09	44.40	47.00	1.06
Olive 270 Bone 0	0.93	0.93	5.80	4.00	1.07	0.82	91.90	104.50	1.14	44.40	49.80	1.12
Tree 0 Bone 0	0.94	0.94	7.80	2.90	0.68	0.61	91.90	102.30	1.11	44.40	48.10	1.08
Tree 90 Bone 0	0.90	0.94	11.90	2.90	1.10	0.57	91.90	109.30	1.19	44.40	49.40	1.11
Tree 180 Bone 0	0.91	0.95	9.60	4.00	1.30	0.63	91.90	104.70	1.14	44.40	47.80	1.08
Tree 270 Bone 0	0.88	0.95	14.90	2.90	1.70	0.58	91.90	113.90	1.24	44.40	48.10	1.08
Mean	0.93	0.93	8.30	4.56	0.95	0.79	91.90	103.92	1.13	44.40	49.83	1.12

The Velocity DIR system uses a B‐spline algorithm and mutual information‐based registration. We also performed DIR tests for materials of different densities, which are listed in Table 4. We selected water, dish soap, and oatmeal to test contours that have a similar density (i.e., dish soap [density = 1.03 g/ml] to DTE oil [density = 0.95 g/ml]) on DIR registration, simulating tumor shrinkage or softening during the course of the radiation treatment. We also tested low‐density material deformation (i.e., oatmeal to oatmeal [density = 0.56 g/ml]) for simulating lung tissue deformation. These results are shown in Table 5.

**Table 4:** List of materials that can be used to fill the cavity

Substance	Density (g/ml)
Air	0
Tobacco, flaked	0.03
Wheat, flaked	0.11
Sesame seeds	0.43
Dry Oatmeal	0.56
Sugar, powdered	0.8
Oval oil	0.91
Corn syrup	0.92
Wax	0.95
DTE oil	0.95
Water	1
Dish soap	1.03
Baking soda	1.12
Sand	1.28
Cort bone	1.7

**Table 5:** Deformable image registration test with materials of different densities

Deformable registration test with different density material			
Reference image (circle) vs second image (tree)	Conformity (Dice)	Surface distance metrics (mm)	
		Max (HD)	Mean
DTE oil (reference)	0.94	7.8	0.6
Water to DTE Oil	0.95	5.8	0.78
Dish soap to DTE Oil	0.94	10.3	0.71
Oat meal to oat meal	0.93	4.9	0.11

## 4 DISCUSSION

To the best of our knowledge, all of the physical phantoms used for DIR testing have either lacked quantitative testing capability or cannot simulate contour deformation and location changes to mimic real patient anatomical changes during the course of treatment. They all demand a great deal of resources and experts to conduct testing. Our new acrylic phantom has a simple design and includes a variety of inserts that are provided to simulate different tissue shapes and properties. As mentioned earlier, this phantom can simulate deformations and location changes in patient anatomy by changing the rotations of both the phantom and the inserts. Both rigid and DIR accuracy can be verified with this single phantom effectively and efficiently to ensure system performance.

It has been reported that the accuracy of DIR algorithms has been tested inconsistently.^17^, ^18^, ^19^, ^20^, ^21^ A multi‐institution study was conducted to provide a consistent and direct comparison of the various algorithms and the performance of different systems.^22^ The report indicated that there were large discrepancies in shifts and the DIR accuracy was equivalent to the voxel size. Our goal in this study was to design a phantom that could be used by any cancer institution that uses DIR in the clinic, either for commissioning or for quality assurance after DIR software upgrades. The phantom is made of acrylic and uses the existing plugs from an RMI phantom, which are available at most cancer treatment centers. It also requires less crafting, making it favorable for mass production at a low cost. We believe it has potential as a prototype phantom for national accreditation purposes to standardize the performance evaluation of all DIR systems across the country.

Multimodality DIR is valuable in surgical planning that requires clear delineation of soft tissue (e.g., spinal cord, cerebrospinal fluid, and nerve bundles). There is also a substantial clinical need for magnetic resonance imaging (MRI) and CT registration in radiation oncology, especially for central nervous system sites. Reaungamornrat et al. used target points from real patient MRIs to test the modality‐independent neighborhood descriptor demons, a deformable registration of MR and CT.^23^ Image registration errors on commercial software using MRI have not been widely studied. Recently, there has been research on the use of synthetic images derived from patient longitudinal deformations and porcine phantoms implanted with markers for MRI DIR accuracy tests.^24^ The phantom that we developed in this study can also be imaged with MRI and includes inserts that can be filled with materials of various densities simulating tissue that has low imaging contrast, i.e., brain. Future work needs to be performed in this area.

The DIR software provided by several vendors provides tools such as CT to CT registration, positron emission tomography‐CT fusion, MRI to CT fusion, custom contour atlas creation, dose deformation, and adaptive re‐contouring. The focus of the Wuphantom design was rigid image registration, DIR, and adaptive re‐contouring accuracy tests. Our phantom can be filled with a solution mixed with F‐18 or Tc‐99 m for positron emission tomography/single‐photon emission CT imaging registration tests or embedded with thermoluminescence dosimeter (TLDs) for dose deformation validation.

There are limitations in the current study. Although the preliminary test results indicate that the Velocity adaptive re‐contouring tool provides reasonably good estimates of contours generated from the original CT image set, the inserts were filled with uniform contents (DTE oil, dish soap, oatmeal, and bone) and contoured accordingly. Actual patient contours will have varied CT# within the volume (i.e., the clinical target volume encompassing the gross tumor and surrounding tissue). As a result, the inserts will need to be tested with mixed contents in the future. Our test used a standard head and neck CT scan protocol for both the reference and secondary images. It should be pointed out that the registration results for both rigid and deformable modalities were not fully evaluated when the scan protocol changed (i.e., noise value changes due to auto mAs use or slice thickness changes). Cone beam CT has been used as a main imaging tool for future online adaptive therapy. The accuracy of cone beam CT vs conventional CT registration would need to be fully studied prior to clinical use. Further work needs to be performed in this area.

## 5 CONCLUSIONS

We have developed a physical phantom that can provide a complete end‐to‐end evaluation of the accuracy of rigid and DIR system. The phantom has defined dimensions and a variety of inserts that can change the shape and contents, simulating different tissue characteristics. The phantom has a low cost; thus, it is widely accessible to clinics throughout the country and world.

## CONFLICT OF INTEREST

The authors declared no conflict of interest.
